# Initiative for ethical media reporting about mental health

**DOI:** 10.1192/j.eurpsy.2022.920

**Published:** 2022-09-01

**Authors:** G. Racetovic, M. Latinovic, G. Cerkez, Z. Stjepanovic

**Affiliations:** 1 Healt Center Prijedor, Community Mental Health Center Prijedor, Prijedor, Bosnia and Herzegovina; 2 Ministry of Health and Social Welfare of the Republic of Srpska, Deputy Minister, Banja Luka, Bosnia and Herzegovina; 3 Federal Ministry of Health, Deputy Minister, Sarajevo, Bosnia and Herzegovina; 4 Institute for Population and Development, Project Of Mental Health In Bosnia And Herzegovina, Banja Luka, Bosnia and Herzegovina

**Keywords:** Media, mental health, Ethics

## Abstract

**Introduction:**

As a part of continuous destigmatization of mental illness and paople with mental disorders significant importance has media reporting, especially in modern times.

**Objectives:**

Media reports about mental health (MH) are still an issue of a great discussion concerning their content especially in ethical matters. Many initiatives in different countries resulted in various changes in attitudes and influenced on this topic. But the image of mental illness as well as psychiatry in general are still burdened with the shadow of stigma.

**Methods:**

To show development of one of initiatives in Bosnia and Herzegovina (BH) supported by policy makers through the creation and broad distribution of recommendations for ethical media reporting about mental disorders.

**Results:**

In 2019 Task Force appointed by both entities’ ministries of health in BH developed an publication with recommendation for ethical reporting about mental health topics with special highlights on specific mental disorders (schizophrenia, depression, suicide, addictions, etc.). It was widely distributed to media and health institutions in the country and was officially adopted as the recommendation of the national Regulatory Agency for Media. Trough five rounds of educational workshops in 2019 and 2021 more than 150 media and mental health professionals were introduced with the publication as well as practical implementation of this recommendations (as is exercises of giving statement).

**Conclusions:**

In BH is developed very useful tool for more quality media reporting about MH topics as one of important ways for reducing the stigma and discrimination of people with mental disorders as well as promotion of good mental health

**Disclosure:**

No significant relationships.

